# The antioxidants dilemma: are they potentially immunosuppressants and carcinogens?

**DOI:** 10.3389/fphys.2014.00245

**Published:** 2014-07-14

**Authors:** Soroush Seifirad, Alireza Ghaffari, Mahsa M. Amoli

**Affiliations:** ^1^Endocrinology and Metabolism Research Center, Endocrinology and Metabolism Clinical Sciences Institute, Tehran University of Medical SciencesTehran, Iran; ^2^Department of Internal Medicine, Tabriz University of Medical SciencesTabriz, Iran

**Keywords:** antioxidants, carcinogens, protective agents, immunosuppressive agents, reactive oxygen species, DNA damage, modeling

## Introduction

Antioxidants and a large number of natural supplements were introduced in recent decades (Lu et al., 2013). It has been hypothesized that antioxidant consumption might reverse oxidative stress which decreases the adverse effects elicited as outcomes of oxidative stress including inflammation, carcinogenesis, and atherosclerosis (Ghorbanihaghjo et al., 2012; Bjelakovic et al., 2014). A large number of studies have been conducted in order to convey evidence for beneficial effects of antioxidants in health and disease (Bjelakovic et al., 2012). However, most of these studies were conducted for a short time interval evaluating limited numbers of targeted biochemical markers both in animal and human studies. Only a small number of prolonged cohort studies have been performed to critically assess the effects of chronic consumption of antioxidants. Unexpectedly, in a recently published systematic review by Bjelakovic et al. on 78 randomized clinical trials on antioxidants supplementation including selenium, β-carotene, vitamin C, vitamin A, and vitamin E, not only have no favorable effects been observed, but additionally, mortality rates have risen (Bjelakovic and Gluud, 2007; Bjelakovic et al., 2012). Surprisingly, it has been shown that antioxidant supplementation may increase the risk of skin malignancy in women (Hercberg et al., 2007). There are also a number of reports, in which a history of longtime supplementation with carotenoids has increased risk of malignancy in smokers and patients with tuberculosis (Albanes et al., 1996; Omenn et al., 1996; Holick et al., 2002; Shiels et al., 2011).

Natural and green products are assumed to be harmless in common belief. The general faith toward the health benefits of products containing antioxidants is partly because of misinformation conveyed by industries manufacturing these food products, which promoted putative overuse of these products as dietary supplements. However, the harmful effects of these products have remained obscured for unknown reasons (Lu et al., 2013). It is crucial to emphasize the harmful effects of the chronic consumption of antioxidants, including potential toxicity, in addition to promotion of potential benefits. According to Watson “the time has come to seriously ask whether antioxidant use much more likely causes than prevents cancer” (Watson, 2013). In this article we have tried to discuss probable mechanisms by which chronic antioxidant consumption provokes adverse effects. We have attempted to depict the role of antioxidants in a comprehensive model of inflammation, oxidative stress, and cancer.

## Natural oxidative pathways

From an evolutionary point of view, oxygen was a toxic component which turned into one of the most important molecules essential for life and a vital agent after millions of years of evolution. However, in certain conditions, its toxic effects still continue to protect more evolved organisms against certain pathogens. Reactive oxygen species (ROS) which play an important role in the innate immune response are a group of molecules and reactive ions and radicals derived from oxygen. Phagocytes mainly comprised of macrophages and neutrophils release a variety of molecules including toxic oxygen radicals in a process undergoing respiratory burst upon activation in response to infectious agents and pathogens. It has been observed that patients with impaired ROS production are critically immune suppressed (i.e., patients with chronic granulomatosis disease); (Segal et al., 2000; Assari, 2006) Therefore, it can be assumed that oxidative stress does not always incite damaging effects. Toxic oxygen-derived products such as superoxide, hydrogen peroxide, singlet oxygen, and hydroxyl radical play a critical role in oxygen-dependent intracellular killing of pathogens, and hence, are key factors within the immune system.

## Oxidative stress: a double-edged sword

ROS could be toxic to both normal and abnormal cells (infected by intracellular pathogens and malignant cells). It has been shown that increased oxidative stress could enhance prevalence of malignancies by direct cellular damage, Seifirad et al. (2012); Lu et al. (2013); Seifirad and Masoudkabir (2013) however, as mentioned above oxidative stress when applied as immune system arms could protect organisms from invading pathogens and malignant cells (Weel et al., 1996).

## Reductive stress: a forgotten enemy

Reductive stress concept has been recently introduced. It seems that although reducing agents provoke many adverse effects and cause damages, this phenomenon has been obscured in the shadow of oxidative stress. Antioxidants could also be categorized as reducing agents.

Antioxidants are rich in weakly-bound electrons and could cause direct DNA damage (Lu et al., 2013). Wang and Nguyen, and their research team have demonstrated that the dissociative electron transfer (DET) of weakly-bound e_pre^−^_ to the guanine base is extremely efficient in inducing chemical bond breaks and subsequent breaking of single and double strand DNA in aqueous solutions (Wang et al., 2009; Nguyen et al., 2011).

It should be noted that the superoxide anion (O_2^−^_) would act as an oxidant and a strong reducing agent. In fact O_2_ has a distinguished electron affinity since O_2^−^_ has no positive electron affinity (Lu et al., 2013). In aqueous media O_2^−^_ act as a strong Bronsted base and donates an electron (i.e., Fenton reaction) (Bhattacharjee et al., 2012; Enami et al., 2014). Hence, O_2^−^_ could induce reductive damage to the cell and DNA (Lu et al., 2013).

Recent studies demonstrate that ROS detoxication and enhanced intracellular antioxidant might be pro-tumorigenic (DeNicola et al., 2011; Perera and Bardeesy, 2011). It has been shown that high intake of tea or coffee that are rich in flavonoids in pregnant women might increase the risk of central nervous system tumors and childhood leukemia (Strick et al., 2000; Paolini et al., 2003; Plichart et al., 2008).

In a very recently published study, Lu et al. showed that natural antioxidant supplements may cause adverse effects in healthy humans, and they may increase the rate of malignancies rather than preventing cancers (Lu et al., 2013). By means of femtomedicine, they compared the toxic effects of green tea extract (GT) epigallocatechin gallate (EGCG) as the main flavonoid in green tea, H_2_O_2_, and Cisplatin on human lung and skin normal cells. Based on the IC_50_values (concentration required to kill 50% of untreated cells), they showed that that EGCG and GT were definitely highly toxic against human skin and lung normal cell and treatment with EGCG decreased the cell survival rate in a dose dependent manner. Surprisingly, they also demonstrated that while both EGCG and O2^−^ have a similar redox potential, the reductive damage induced by O2^−^ must be much less than EGCG. According to their results, treatment with EGCG/GT slightly increased the survival rate of lung cancer cells at low concentrations (#100/150 mM), despite the fact that treatment with very high concentrations (100–400 or 150–500 mM) showed some therapeutic effects. This observation was also in accordance with the previous findings (Yang et al., 1998, 2000; Elbling et al., 2005). It should be noted that although extremely high levels of EGCG/TG (100–500 mM) can destroy cancerous cells via the reductive mechanism and DNA damage induction, normal cells are also threatened to be severely damaged at such high level of antioxidants treatment. Therefore, it might be suggested that EGCG/antioxidant supplementation might not have beneficial effects for patients undergoing chemotherapy. In another words, antioxidants may diminish the ability of an exogenous reducing agent in killing tumor cells (Watson, 2013).

## Antioxidant pathways

**Figure 1:**
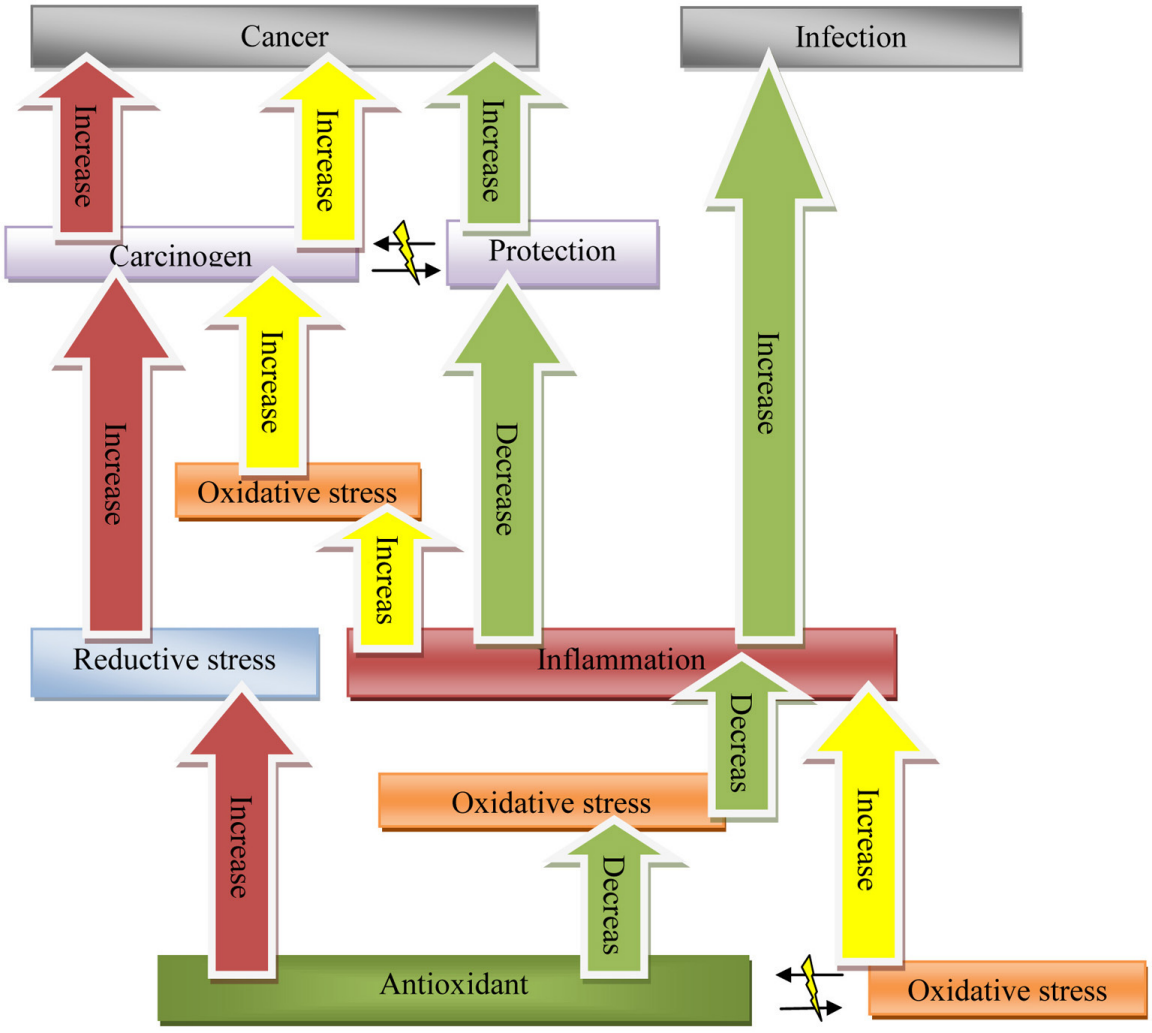
**A comprehensive model for oxidative stress, antioxidants, inflammation, and carcinogenesis: oxidant-antioxidant equilibrium, cancer protection, and carcinogenesis equilibrium are described as determinant balancing points between health and disease states in this model**. Red: Antioxidants as reductive agent. Yellow: Oxidative stress carcinogenesis pathway. Green: Antioxidant as immunosuppressant.

As it has been illustrated in Figure 1:

1. Antioxidants decrease oxidative stress and oxidative damage and subsequently diminish likelihood of developing malignancies due to oxidative stress. This pathway has been the foundation of current thoughts leading to the antioxidant general administration and usage in common belief.
2. Antioxidants could impair function of immune system by means of decreased oxidative stress. This might increase prevalence of malignancies and infections (Figure 1 following green pathway).
3. Since antioxidants are rich in weakly-bound electrons, they could cause direct DNA and cell damage. Hence they could be carcinogens (Figure 1 following red pathway).

## Equilibrium and homeostasis

**Figure 2:**
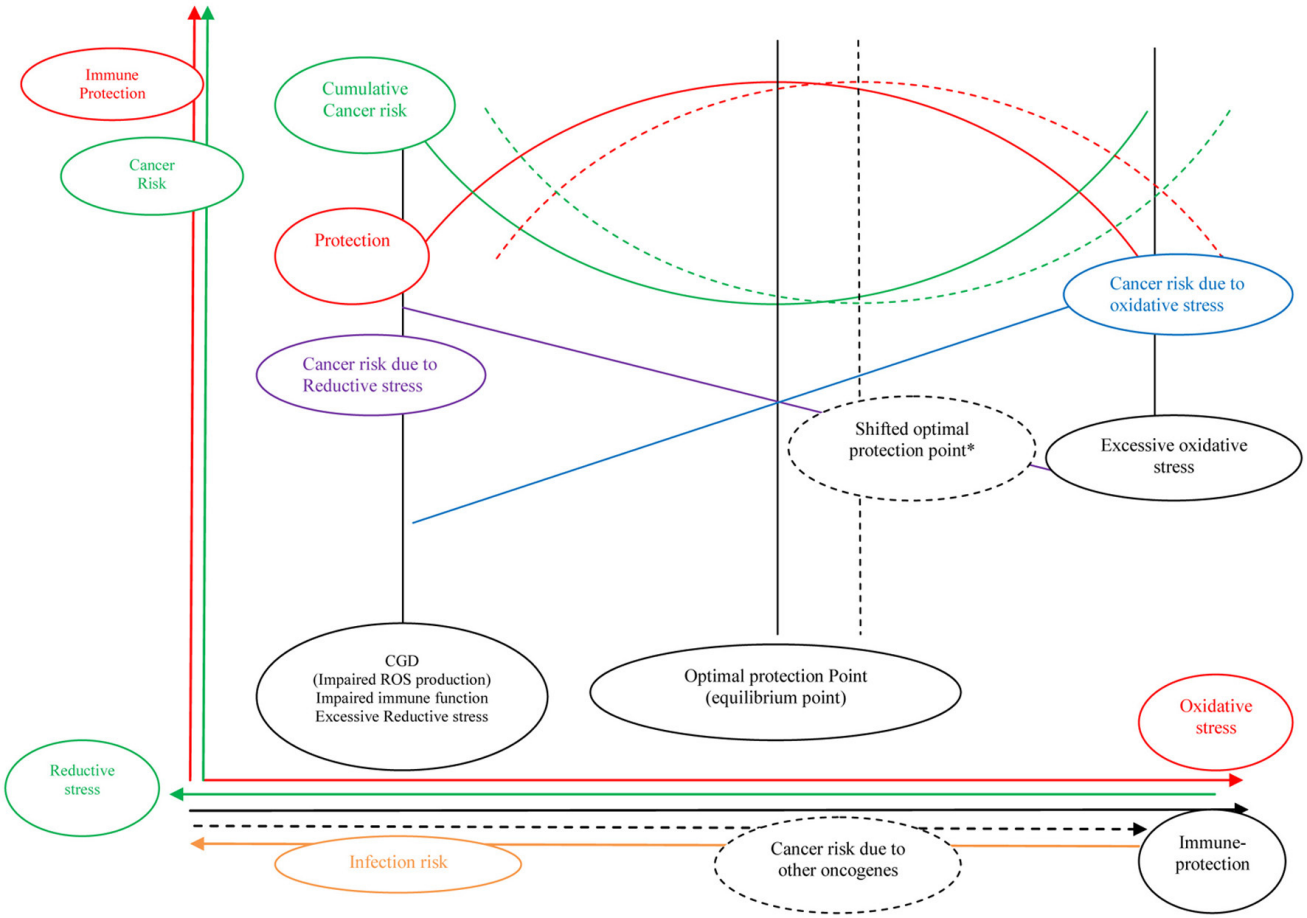
**There is no unit and all figures are representing increase and decrease in parameters, curves, and arrows are representing hypothetical relationship between parameters**. ^*^Both high levels of ROS and antioxidants are carcinogen; however it seems that in certain conditions such as tuberculosis and smoking slightly increased ROS production could be beneficial rather than hazardous. Optimal equilibrium point is shifted in these conditions to the right. Increased risk of cancer due to other oncogenes (i.e., smoking) needs more alert immune system and more oxidative stress, hence optimal protection point will shift to the right.

Traditionally disequilibrium in natural homeostasis is defined as disease state. In traditional medicine, diagnosis and treatment of disorders was based on managing equilibrium (Shahabi et al., 2008). Therefore, when there is equilibrium between oxidant/antioxidant pathways maximum protection is anticipated (health state). Disequilibrium in these pathways could impair defense mechanism and increase likelihood of developing malignancies and infections (Figure 2).

Here we demonstrate some examples to further discuss the effect of imbalance in antioxidant equilibrium in various conditions.

## Smoking, tuberculosis, and antioxidants

Previous studies have observed an increased rate of lung cancer in smokers who were on long time supplementation with β-carotene as well as in patients with tuberculosis treated with carotenoids (Albanes et al., 1996; Omenn et al., 1996; Holick et al., 2002; Shiels et al., 2011). First of all, β-carotene in high levels and chronic consumption could be carcinogenic by means of direct DNA damage; additionally, it might be hypothesized that a slightly increased level of oxidative stress could be protective in smokers and patients with tuberculosis, while a very large increase in oxidative stress could be carcinogenic rather than protective. In fact, it seems that in these cases, the optimal point of oxidative stress is enhanced in order to compensate for extrinsic effects of carcinogens or pathogens (Shifted optimal protection point, Figure 2). Taken together while alert immune system could be protective, antioxidants might down-regulate immune system, by means of decreased inflammation and cell damage.

## Discussion

Industrialization in the recent century significantly affected the human lifestyle. As the transformation in lifestyle and habits has been so rapid, it has not been attenuated as a matter of evolution by means of time. Modern human beings began smoking, as well as producing and consuming food colorings, food preservatives, and fast foods. Medicine has been developed and a large number of antibiotics and oxidant agents (i.e., alkaline agents, antibiotics, and other chemotherapy agents for anticancer treatment) were produced. On the other hand, there has been an effort by scientists to transverse these effects by exogenous artificial manipulations and interventions like antioxidant supplementation. However, the limited knowledge of complex physiology of the human system has resulted in further complications (Seifirad, 2014). Lack of appropriate knowledge and the complexity of systems and physiological pathways has exacerbated troubles of industrialized society, which is beyond the simplified approach currently utilized in resolving complex dilemmas. Broad antioxidant treatment has been one of these manipulations that might be harmful rather than beneficial if prescribed without precise consideration. In fact, in the average human diet, there would be no need for such a high consumption of supplements including antioxidants.

In conclusion, oxidative stress and inflammation are not as harmful as it has been assumed. They are natural defense systems in the human body working against infectious diseases as well as malignancies. Oblivious antioxidant therapy could be harmful rather than beneficial for health. Excessive antioxidant therapy could impair immune system and increase likelihood of developing malignancies by means of decreased immune protection and direct cell and DNA damage.

## Suggestion

Studies on modeling and determining optimal oxidative stress point are warranted.

### Conflict of interest statement

The authors declare that the research was conducted in the absence of any commercial or financial relationships that could be construed as a potential conflict of interest.
